# How countries’ legislations can sustainably impact children’s mental health

**DOI:** 10.1192/j.eurpsy.2023.1532

**Published:** 2023-07-19

**Authors:** M. R. Moro, A. Ogrizek, E. Dozio, D. Attias, G. Papazian-Zohrabian, J.-M. Jesus-Martin, M. Teicher, T. Baubet

**Affiliations:** 1 Child psychiatry, Hopital Cochin, Maison de Solenn; 2 Psychologist, Action contre la faim, Paris, France; 3 Juvenile attorney, executive board of the European lawyers’ foundation, La Haye, Netherlands; 4 Psychologist, Université de Montréal, Montréal, Canada; 5 Child psychiatry, Baylor College of Medicine; 6 Biopsychiatry, Harvard Medical School, Boston, United States; 7 Child psychiatry, Université Sorbonne Paris Nord, Paris, France

## Abstract

**Introduction:**

In a new era where, more and more children are standing up against governments concerning important subjects like climate change that will impact their physical health in a near future, it is time to question ourselves on all the other decisions that are being taken and that could have a sustainably high impact on some of our children’s development and mental health. Unfortunately, many of those children are forced to remain silent - unable to express themselves - or are just not being heard – unable to gain international medias’ attention - because of their social condition, cultural background, age or religion. But more sadly, most of them remain silent because they are just unaware of the consequences their living conditions or hardships might have on their future mental health, due to lack of information or education.

**Objectives:**

Therefore, it is our responsibility as childhood experts and professionals to speak for those children who cannot, to stand up for themselves and promote the importance of putting their interest first no matter what.

**Methods:**

We have chosen six different studies led in different contexts of struggle for children all around the world to illustrate the consequences on their development and mental health.

**Results:**

We will communicate on the situations of children living in refugee camps, children living with their mothers in prison cells, children being forcibly separated from their mothers returning from Daesh territories in France or children being forcibly separated from their migrant mothers at the US border, we will describe the hardships but also the effective support provided to unaccompanied minors in Canada, and especially discuss with our cochair expertise how the issue is or could be different for them according to government policies and legislations.

**Conclusions:**

By describing these different contexts of unstable living conditions or traumatic experiences orchestrated by government legislation regarding children care, we want to highlight the responsibility that every government legislation must consider when it comes to child care and how it should become an absolute priority.

**Disclosure of Interest:**

None Declared

